# PRMT1 regulates the tumour-initiating properties of esophageal squamous cell carcinoma through histone H4 arginine methylation coupled with transcriptional activation

**DOI:** 10.1038/s41419-019-1595-0

**Published:** 2019-05-01

**Authors:** Yue Zhao, Qijue Lu, Chunguang Li, Xinyu Wang, Long Jiang, Lei Huang, Chao Wang, Hezhong Chen

**Affiliations:** 1 Department of Thoracic Surgery, Changhai Hospital, Second Military Medical University, 200433 Shanghai, China; 2 Department of Urology, Changhai Hospital, Second Military Medical University, 200433 Shanghai, China

**Keywords:** Cancer stem cells, Cell signalling

## Abstract

Esophageal squamous cell carcinoma (ESCC) is the most difficult subtype of esophageal cancer to treat due to a paucity of effective targeted therapy. ESCC is believed to arise from tumour initiating cells (TICs), which contribute to metastasis and chemoresistance. In this study, we found that Protein arginine methyltransferase 1(PRMT1) was highly expressed in ESCCs and associated with aberrant clinicopathological characteristics of ESCC patients. In ESCC specimens, the elevated expression of PRMT1 and OV6 was significantly associated with histologic grade, TNM stage and poor patient prognosis. Moreover, overexpression of PRMT1 was observed in esophageal TICs purified by magnetic sorting of adherent and spheroid ECA109/TE1 cells. The increased level of PRMT1 in TICs facilitated the expression of TIC markers, stem cell-like properties, resistance to chemotherapy, tumorigenicity and increased their percentages in ECSS samples. Conversely, knockdown of PRMT1 significantly diminished the self-renewal properties of ESCC. Moreover, we show that PRMT1 can catalyse histone H4R3 asymmetric dimethylation and promote transcription activation of down-stream genes. Further RNA-Seq transcriptome analysis reveals that overexpression of PRMT1 in ESCC cell lines activates Wnt/β-catenin and Notch signaling pathway. Together, our studies highlight that PRMT1 activates and maintains esophageal TICs by mediating transcription alteration through histone H4 arginine methylation.

## Introduction

As a common malignant cancer, esophageal cancer (EC) ranks sixth in the cause of cancer-related death all over the world^1^. Esophageal squamous cell carcinoma (ESCC), the major histopathological type of esophageal cancer, is an aggressive tumour that is characterized by early metastasis and late diagnosis^2^. Although treatments for ESCC have made progress in the past few decades, the overall survival of advanced ESCC patients remains poor, even after surgery^3^. Therefore, recent ESCC research focuses on understanding the molecular mechanisms of ESCC initiation and progression as well as identifying novel potential therapeutic targets.

The significance of tumour initiating cells (TICs) was first identified in leukemia and soon reported in various solid tumours, such as breast, prostate and pancreatic cancers^4,5^. Increasing experimental data indicated that TICs have the ability to facilitate the initiation of tumour growth as well as contribute to tumour treatment resistance. Recently, studies reported that several cell surface markers in ESCC TICs have been identified, including CD133, CD44 and CD90^6–8^. OV6, an epithelial origin marker, was initially verified in hepatocellular carcinoma TICs and associated with stem cell-like properties, including tumorigenicity, recurrence, chemoresistance and metastasis^9^. Our previous study identified OV6 as a novel esophageal TIC marker, because the OV6^+^ ESCC cells exhibit the ability to form tumours and possesses a stronger resistance to chemotherapy^10^. However, the underlying mechanisms that regulate the expansion and function of TICs in ESCC remain unclear.

Protein arginine methylation is one of the most important post-translational modifications (PTMs); ~2% of arginine residues in proteins of nuclear extracts are methylated^11^. Protein arginine methyltransferase (PRMT) is a methyltransferase that catalyses the methylation of histones and non-histone proteins from S-adenosylmethionine (SAM) to arginine residues. Many cellular processes involve these modified proteins, such as signal transduction, DNA repair etc.^12^. Nine members of the PRMT family have been identified and can be divided into three categories based on the methylation mark deposited on the arginine residue: the type I family of enzymes (PRMT1, PRMT2, PRMT3, PRMT4/CARM1, PRMT6, PRMT8), the type II enzymes (PRMT5, PRMT9), and the type III enzymes (PRMT7)^13^. Furthermore, arginine can either be mono-methylated or di-methylated, the latter of which can be either symmetric or asymmetric depending on the type of protein methyl transferases depositing the mark^14^. Importantly, proteins that contain arginine-glycine-rich motifs (RG) are often targets for PRMT-mediated methylation.

PRMT1, a member of the type I family of arginine methyltransferase, is the most abundant PRMT and is responsible for nearly 75% of all arginine methylation in cells^15^. It has been reported that two types of methylation are mediated by PRMT1, including monomethylation and asymmetric dimethylation^16^. Moreover, PRMTs were initially found to directly methylate various histone proteins to regulate different cellular functions. Among these PRMTs, PRMT1 is predominantly known to catalyze methylation of histone H4R3 and correlate with activated transcription^17,18^.

A growing amount of evidence indicates that arginine methylation might be a driver for the initiation and progression of cancer^19^. Dysregulation of PRMT1 has been observed in several cancer types. PRMT1 was reported to be upregulated in breast cancer, lung cancer and colon cancer and to promote the proliferation and transformation of cancer cells^11,20,21^. However, the roles of PRMT1 in ESCC are not well understood. Furthermore, PRMT5 plays an important role in the maintenance of breast TICs^22^. Indeed, PRMT5 has also been shown to be responsible for leukemic and glioblastoma stem cell function^23,24^. The role of PRMT5 in tumour initiating cell function might indicate a role of the PRMT family in regulating the self-renewal capacity of cancer cells. Nevertheless, there is a limited understanding of PRMT1, the most abundant member of the PRMT family, in the regulation of TICs.

In this study, we investigated the role of PRMT1 in regulating the initiation and development of ESCC. We showed that PRMT1 is predominantly expressed in ESCC cell lines and patients, and that its expression level was closely associated with aberrant clinical pathological features and poor patient prognosis. Moreover, we also verified that PRMT1 is preferentially expressed in esophageal TICs and functions to enhance the self-renewal features, tumorigenicity and chemoresistance of ESCC. Moreover, PRMT1 upregulated the expression of histone H4R3 asymmetric dimethylation (H4R3me2a), which might be activated to stimulate transcriptional regulation of downstream target genes^25^. Moreover, transcriptome analysis by RNA-seq reveals that PRMT1 overexpression leads to activation of Wnt/β-catenin signaling pathway and Notch pathway. In conclusion, PRMT1 as a novel effector promotes the self-renewal properties in esophageal TICs during the progression of ESCC.

## Material and methods

### Patients and tissue specimens

Ninety-five patient specimens were collected following radical esophagus surgery in 2012 and 2013 from the Department of Thoracic Surgery, Changhai Hospital, Shanghai, China. All samples were fixed in 4% formaldehyde and embedded in paraffin wax. The patient samples were obtained with informed consent according to an established protocol approved by the Ethics Committee of Changhai Hospital. All patients were observed until May 2017, with a median observance time of 27 months.

### Cell culture

Eca109, TE1 and HEEC cells were obtained from the Shanghai Cell Bank (Shanghai, China). After measured by mycoplasma detection, DNA-Fingerprinting, isozyme detection and cell vitality detection, these cell lines were immediately expanded and frozen such that they could be restarted every 3–4 months. All cell lines maintained in Dulbecco’s modified Eagle’s medium (Gibco, CA, USA) supplemented with 10% heat-inactivated foetal bovine serum (Gibco-BRL) and antibiotics (100 U/ml penicillin and 100 U/ml streptomycin; HyClone Laboratories, Inc., USA) at 37 °C in a humidified atmosphere of 5% CO2. Spheroids were cultured in F12/DMEM (Gibco, CA, USA) supplemented with EGF (Sigma, St Louis, USA), FGF (Gibco, CA, USA) and ITS (Sigma, St Louis, USA).

### Establishment of PRMT1 overexpression and knockdown cells

We constructed lentiviral vectors encoding the human PRMT1 gene or green fluorescent protein (GFP) in the pLenti-CMV-PGK-EGFP-T2A-Puro vector (HeYuan Bio-technology Co., Shanghai, China) and designated them as LV-PRMT1 or LV-GFP. The lentiviral vectors were transfected into the HCC cells with a multiplicity of infection (MOI) 5 in the presence of polybrene (5 μg/ml) for 6 h. Stable Eca109 and TE-1 cells knockdown of PRMT1 were generated using lentiviral constructs expressing shPRMT1(shPRMT1^1#^ GGACATGACATCCAAAGATTA; shPRMT1^2#^ GCAACTCCATGTTTCATAACC; shPRMT1^3#^ GCAACTCCATGTTTCATAACC) and negative control (HeYuan Bio-technology Co., Shanghai, China), and incubated with 2 μg/ml puromycin (Sigma, St Louis, USA).

### Magnetic cell sorting and flow cytometry

Cells were labelled with primary OV6 antibody (mouse IgG1; R&D Systems, Minneapolis), magnetically tethered to rat anti-mouse IgG1 microbeads, and sorted with a Mini-MACS™ Cell Sorter Kit (Miltenyi Biotec, CA). All of the procedures were following the manufacturer’s instructions. The sorted cells were evaluated by flow cytometry analysis. The flow cytometry was performed with MoFlo Sorter (Beckman, Brea, CA) using an APC-conjugated-OV6 antibody (R&D Systems, Minneapolis) and following manufacturer’s instruction.

### TICs spheroid formation assay

Cells were magnetically sorted, then 3000 OV6^+^ cells were cultured in Ultra-Low Attachment six-well plates (Corning Lnc., Coring, NY) for 10 days. The number of spheroids was measured under microscopy and the representative pictures were taken. All experiments were performed in triplicates.

### Quantitative real-time polymerase chain reaction (qRT-PCR)

Total RNA was extracted from cultured ESCC cell lines or magnetic sorted cells using Trizol (Invitrogen, Grand Island, NY) according to the manufacturer’s instruction. The cDNA was synthesized using the PrimeScript RT Reagent Kit (TaKaRa Bio, Shiga, Japan) following the manufacturer’s instructions. Real-time PCR was performed on a Roche Light Cycler 480 (Roche) using SYBR Green PCR Master Mix (TaKaRa Bio, Shiga, Japan). Primer sequences are listed in Table 1. Each measurement was performed in triplicates and the results were normalized by the expression of the GADPH gene. Fold change relative to mean value was determined by 2^−ΔΔCt^. All experiments were performed in triplicates.

**Table 1:** Sequence of PCR primers used in this study

Gene	Forward primer sequence (5′-> 3′)	Reverse primer sequence (5′-> 3′)
CD133	GCCACCGCTCTAGATACTGC	TGTTGTGATGGGCTTGTCAT
CD44	GAGCATCGGATTTGAGA	CATACTGGGAGGTGTTGG
ABCG2	CAGGTTACGTGGTACAAGATGA	GATCAGTGATAAGCTCCATTCC
KLF4	CCATTACCAAGAGCTCATGC	GTGCCTGGTCAGTTCATCTG
SOX2	CAAGATGCACAACTCGGAGA	GCTTAGCCTCGTCGATGAAC
NANOG	CTGCTGGACTGAGCTGGTTGCC	GCTGAGGCCTTCTGCGTCACA
OCT4	AGTGAGAGGCAACCTGGAGA	ACACTCGGACCACATCCTTC
PRMT1	CTTTGACTCCTACGCACACTT	GTGCCGGTTATGAAACATGGA
EPCAM	AATCGTCAATGCCAGTGTACTT	TCTCATCGCAGTCAGGATCATAA
CXCR4	ACTACACCGAGGAAATGGGCT	CCCACAATGCCAGTTAAGAAGA
c-MYC	GTCAAGAGGCGAACACACAAC	TTGGACGGACAGGATGTATGC
β-catenin	GGCCCAGAATGCAGTTCGCCTT	AATGGCACCCTGCTCACGCA
FZD10	AGCCATCCAGTTGCACGAG	GAGTCGGGCCACTTGAAGTT
RAC3	TCCCCACCGTTTTTGACAACT	GCACGAACATTCTCGAAGGAG
DLL3	CACTCCCGGATGCACTCAAC	GATTCCAATCTACGGACGAGC
EGFL7	TGAATGCAGTGCTAGGAGGG	GCACACAGAGTGTACCGTCT
CCND1	GCTGCGAAGTGGAAACCATC	CCTCCTTCTGCACACATTTGAA
LGR5	CTCCCAGGTCTGGTGTGTTG	GAGGTCTAGGTAGGAGGTGAAG
RUNX2	TGGTTACTGTCATGGCGGGTA	TCTCAGATCGTTGAACCTTGCTA
NOTCH1	GAGGCGTGGCAGACTATGC	CTTGTACTCCGTCAGCGTGA
NOTCH2	CAACCGCAATGGAGGCTATG	GCGAAGGCACAATCATCAATGTT
JAGGED1	GTCCATGCAGAACGTGAACG	GCGGGACTGATACTCCTTGA
HEY-1	GTTCGGCTCTAGGTTCCATGT	CGTCGGCGCTTCTCAATTATTC
HES-1	TCAACACGACACCGGATAAAC	GCCGCGAGCTATCTTTCTTCA

### Total proteins and histone extraction and western blotting

Whole cultured cells were homogenized in 0.1% SDS and 1 mM PMSF (phenylmethylsulfonyl fluoride) and centrifuged at 12,000 g for 15 min. The total histone proteins were extracted from ESCC cells using a Histone Extraction Kit (Abcam, ab113476), following manufacturer’s protocol. Protein extracts were subjected to SDS-PAGE and analysed using the following primary antibodies: PRMT1 (Abcam, ab73246), H4R3me2a (Active Motif,), H4R3me2s (Active Motif), histone H3 (CST) and GADPH (Abcam, ab8245). Then the membranes were incubated with secondary antibodies (CST,7076,7074) at room temperature for 1 h. The dilution ratio was determined according to the recommended instructions. Protein levels were detected by the Image-Pro Plus 6.0 system (Bio-Rad,1708265). Quantification of bands intensity was measured using ImageJ software (version 1.34). All experiments were performed in triplicates.

### Immunohistochemistry

95 ESCC tissues were fixed in 4% methanol, embedded in paraffin, and cut into a thickness of 5 µm. After deparaffinization and rehydration procedures, antigen recovery was performed in a heated citrate buffer (pH 6.0) or EDTA buffer (PH 8.0) for 30 min. Then, the slides were incubated with UltraSensitive Streptavidin Peroxidase Kit (Fuzhou Maixin Biotechnology, Fuzhou, China) and the anti-OV6 (1:50; R&D systems, Minneapolis, MN) and anti-PRMT1 (1:200, Abcam, ab73246) primary antibodies at 4 ℃ overnight. Then, diaminobenzidine (DAB) (Dako, Carpinteria, CA, USA) staining was used to image specific markers. The immunostaining staining scores were evaluated as 0, 1 (low expression) and 2, 3 (high expression).

### Chemotherapy drug treatments, soft agar colony formation assay, and viability assay

ECA109 and TE1 cells from different groups were treated with cis-platinum (2.0 g/mL) for 4 days, and the percentage of OV6^+^ cells was then measured by flow cytometry. For the soft agar assay, magnetically sorted OV6^+^ cells were cultured in 1 mL of 0.7% agarose with DMEM-mixed upper gel on six-well plates. Then, 0.6 g/mL of cis-platinum was added into six-well plates and incubated for 2 weeks. Colony formation numbers was determined by microscope counting. Cell viability after drug treatment was assessed by a cholecystokinin-8 assay (CCK8).

### Xenograft mice

Six-week-old male nonobese diabetic/severe combined immunodeficient (NOD/SCID) mice were purchased from Shanghai Experimental Center (Shanghai,China). Mice were used to evaluate the effects of PRMT1 upregulation and downregulation of on tumorigenicity and tumour growth. Briefly, different numbers of Eca109 OV6^+^ cells were suspended in 200 µL of DMEM and Matrigel (1:1) (Corning) and injected into the subcutaneous tissue of NOD/SCID mice. Tumour size and incidence were measured weekly. Mice were sacrificed at the indicated time points according to the protocols approved by the SMMU Animal Care Facility and the National Institutes of Health guidelines. Tumours were harvested for assessment of tumour size, tumour incidence, western blot and immunohistochemistry analysis.

### RNA-seq transcriptome analysis

RNA-seq was performed on three biological replicates. Total RNA from ECA109 LV-GFP/LV-PRMT1 was extracted by Trizol and kept at −80 °C. DNA was removed using a DNA-free DNA Removal Kit (Thermo Fisher, AM1906). The RNA quantitation and quality measurement were checked using Bioanalyzer 2200 (Agilent Technologies, USA). RNA with a RIN (RNA integrity number) >6.8 was considered acceptable for cDNA library construction. TruSeq Stranded mRNA Library Prep Kit (Illumina, Inc.) was used to construct cDNA libraries for each RNA sample according to the manufacturer’s instructions. RNA-Seq bioinformatic analysis were performed by Shanghai Novel-bio Company. Before read mapping, clean reads were obtained from the raw reads by removing the adaptor sequences and low-quality reads. The clean reads were then aligned to human genome (GRCh38, NCBI) using the Hisat2^26^. HTseq was used to get gene counts and RPKM method was used to determine these gene expression^27^. Then, we applied DESeq2 algorithm^28^ to filter the differentially expressed genes, after the significant analysis, *P*-value and FDR analysis were subjected to the following criteria: (a) Fold Change > 2 or < 0.5; (b) *P*-value < 0.05, FDR < 0.05.

Gene ontology (GO) analysis was performed to facilitate elucidating the biological implications of the differentially expressed genes in the experiment. We downloaded the GO annotations from NCBI (http://www.ncbi.nlm.nih.gov/), UniProt (http://www.uniprot.org/) and the Gene Ontology (http://www.geneontology.org/). Fisher’s exact test was applied to identify the significant GO categories (*P*-value < 0.05).

Pathway analysis was used to find out the significant pathway of the differentially expressed genes according to KEGG database. We turn to the Fisher’s exact test to select the significant pathway, and the threshold of significance was defined by *P*-value < 0.05. All RNA-Seq files are available from the GEO database (accession number: GSE128913).

### Oncomine and CCEL analysis

The expression levels of PRMT1 in ESCC sample were detected through analysis in Oncomine database (www.oncomine.org). Student’ *t* test was used to calculate statistical significance between ESCC specimens and normal specimens. The fold change was set up at 2 and the threshold of significance was defined by *P*-value < 0.01. Meta-analysis was used to evaluate PRMT1 gene expression from two ESCC Oncomine database. The colored squares indicated the median rank for PRMT1(vs. Normal tissue) across two analyses. The mRNAs expression level of PRMT1 in a series of cancer cell lines were determined by CCLE database (https://portals.broadinstitute.org/ccle/home). The CCLE is an online database of more than 1100 human cell lines, which was used to detect gene expression and large-scale parallel sequencing data.

### Statistical analysis

Analysis was performed with SPSS software. Qualitative variables were compared by the chi-square test of Fisher’s exact test, and quantitative variables were measured by the *t* test, paired Wilcoxon signed rank test or Spearman rank correlation test. One-way analysis with ANOVA was used to analyse significant differences between the groups. Kaplan–Meier and Log-rank test analysis was used to determine survival. All data are presented as the mean ± SD. All statistical tests were two-sided, and *P* < 0.05 was considered to indicate statistical significance.

## Results

### Increased expression of both OV6 and PRMT1 is significantly related with aberrant clinicopathological characteristics and poor prognosis in patients with ESCC

**Fig. 1:**
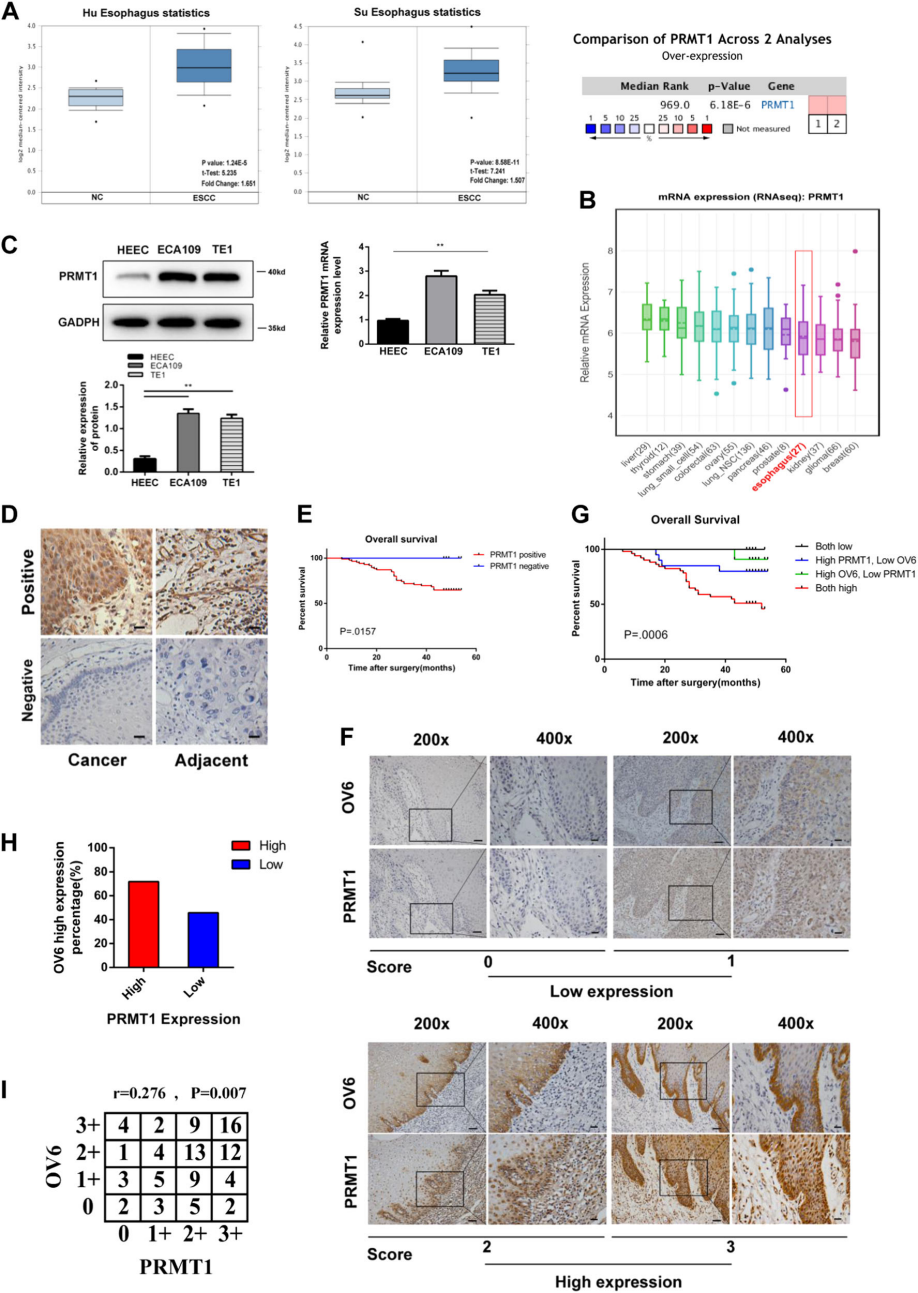
PRMT1 is predominantly expressed in ESCC and positively associated with OV6 expression. **a**, **b** ONCOMINE (**a**) and CCLE (**b**) data showed that PRMT1 was highly expressed in ESCC patients and cell lines. **c** Western blot and qRT-PCR data showed the elevated PRMT1 expression in ECA109 and TE1 cell lines compared with HEEC cells. **d** PRMT1 expression in ESCC cancer tissue compared with adjacent animal tissue (scare bar = 20 µm). **e** Overall survival curve of patients with negative PRMT1 expression (blue line) and patients with positive PRMT1 expression (red line), *P* = 0.0157. **f** 95 ESCC patient samples were used for immunohistochemistry analysis of the expression of PRMT1 and OV6. Representative staining intensity of PRMT1 and OV6 expression represents different expression level (×200, scale bar = 100 µm; ×400, scale bar = 20 µm). **g** The overall survival rates of 95 patients with ESCC were compared among different groups, *P* = 0.0006. **h**, **i** The percentage of patients with high OV6 staining is higher in the PRMT1-high group (upper panel). Spearman correlation analysis shows a significant correlation of the expression levels of PRMT1 and OV6 in ESCC (lower panel), *P* = 0.007 *r* = 0.276

Previous research from different groups have shown that PRMT1 is elevated to variable extents in many kinds of cancers^17^. Here, we verified the increased expression of PRMT1 in ESCC and the correlation of PRMT1 with patient clinicopathological characteristics. To begin, the ONCOMINE and CCLE data showed that the expression level of PRMT1 was elevated in tumour patients and cell lines of ESCC (Fig. 1a, b). To further confirm the expression of PRMT1, we examined PRMT1 expression by western blot and qRT-PCR analysis. The PRMT1 expression level was obviously upregulated in the ESCC cell lines compared with the HEEC normal esophageal epithelium cell line (Fig. 1c). Next, we examined PRMT1 expression by immunohistochemistry analysis in ESCC and adjacent normal tissue. The positive expression of PRMT1 accounted 89.5% of patients with ESCC, while only 46.3% in adjacent normal tissue (Fig. 1d and Table 2). Moreover, the positive staining of PRMT1 in ESCC was significantly correlated with aberrant clinicopathological characteristics and poor prognosis of ESCC patients (Table 3, Fig. 1e). Our previous study illustrated that OV6, a novel TIC biomarker, was increased in ESCC and was closely correlated with clinical pathological features and poor patient prognosis^10^. In this study, we further explored a possible correlation of PRMT1 and OV6 expression in 95 patients through immunohistochemistry analysis (Fig. 1f). Based on expression level, these patients were classified into four groups: group I (*n* = 51), high OV6 and PRMT1 intensity; group II (*n* = 20), high PRMT1 but low OV6 intensity; group III (*n* = 11), high OV6 but low PRMT1 intensity; and group IV (*n* = 13), low OV6 and PRMT1 intensity. In the PRMT1 high-expression groups, the percentage of patients with high OV6 expression was 71.8%, higher than that in the OV6 low-expression groups (45.8%), showing an association between OV6 and PRMT1 expression (Fig. 1h). Moreover, patients with high expression of both OV6 and PRMT1 possessed aggressive clinicopathological features, including high T grades and TNM stage (Table 4). Additionally, the association between OV6 and PRMT1 expression in the same tissue by using Spearman’s rank correlation indicated that the aberrant expression of OV6 was positively related to the overexpression of PRMT1 in ESCC tissue (Fig. 1i). More importantly, the differences in overall survival among the four groups were significant (Fig. 1g). Therefore, the combination of OV6 and PRMT1 expression can be an independent prognostic predictor for ESCC.

**Table 2:** Expression of PRMT1 in ESCC tumour tissues and corresponding adjacent normal tissues

PRMT1 expression, *n* (%)				
	Cases	Negative	Positive	*P* value
Tumour tissues	95	10 (10.5)	85 (89.5)	**<0.01**
Normal tissues	95	51 (53.7)	44 (46.3)	

The bold figure in tables means p-value is significant

**Table 3:** Correlation between PRMT1 expression and clinicopathological characteristics in 95 patients

	PRMT1		
	Negative (10)	Positive (85)	*P* value^a^
*Sex*			
Male	9	69	0.684
Female	1	16	
*Age (years)* ^b^			
>65	2	23	0.727
≤65	8	62	
*T grade*			
1	7	21	**0.005**
2	3	22	
3	0	40	
4	0	2	
*Lymph node metastasis*			
N0	8	49	0.406
N1	2	19	
N2	0	13	
N3	0	4	
*TNM stage*			
I	4	27	**0.028**
II	6	26	
III	0	32	
*Death*			
yes	0	30	**0.028**
no	10	55	

^a^Statistical significance was determined by chi-square test or Fisher’s exact test

^b^Data are presented as the mean ± S.D

The bold figure in tables means p-value is significant

**Table 4:** Clinicopathological characteristics by OV6 and PRMT1 expression

OV6/PRMT1 expression					
	Both high (51)	High-PRMT1 Low-OV6 (20)	High-OV6 Low-PRMT1 (11)	Both Low (13)	*P* value^a^
*Sex*					
Male	43	16	9	10	0.909
Female	8	4	2	3	
*Age (years)* ^b^					
>65	13	5	3	4	0.938
≤65	38	15	8	9	
*T grade*					
1	10	9	3	6	**0.005**
2	11	8	5	1	
3	29	2	3	6	
4	1	1	0	0	
*Lymph node metastasis*					
N0	25	15	6	11	0.437
N1	13	3	4	1	
N2	9	2	1	1	
N3	4	0	0	0	
*TNM stage*					
I	10	12	4	7	**0.018**
II	17	5	4	4	
III	24	3	3	2	
*Death*					
yes	25	4	1	0	**<0.001**
no	26	16	10	13	

^a^Statistical significance was determined by chi-square test or Fisher’s exact test for categorical/binary measures and by ANOVA for continuous measures

^b^Data are presented as the mean ± S.D

The bold figure in tables means p-value is significant

### PRMT1 is predominantly expressed and increases stem cell-like properties in esophageal TICs

**Fig. 2:**
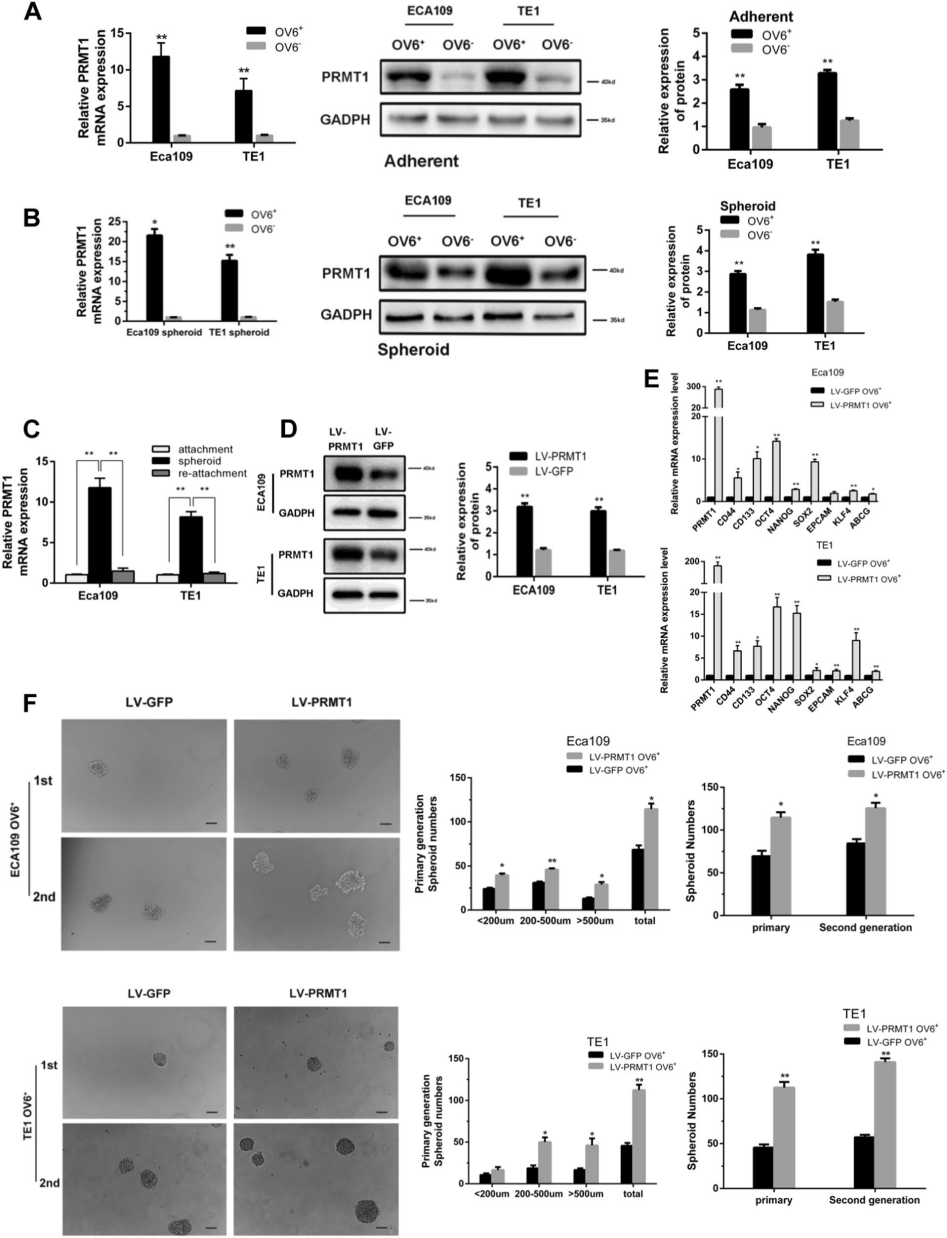
PRMT1 is predominantly expressed in esophageal TICs and promotes tumour initiating cell-like properties of TICs. **a**, **b** qRT-PCR and western blotting analysis of magnetically sorted OV6^+^ adherent (**a**) and spheroid (**b**) subpopulations was performed to evaluate the relative mRNA and protein expression levels, respectively, of PRMT1 in ECA109 and TE1 cells. Data are shown as the mean ± SD, *P < 0.05, **P < 0.01. **c** qRT-PCR analysis of PRMT1 expression of ECA109 and TE1 cells in different culture conditions. Data are shown as the mean ± SD, **P < 0.01. All experiments were performed in triplicates. **d** The protein level of PRMT1 in the LV-PRMT1 group was significantly upregulated in ECA109 and TE1 cells compared with the LV-GFP group. **e** qRT-PCR analysis was performed for PRMT1 and the stem cell-associated genes in magnetically sorted LV-PRMT1 OV6^+^ or LV-GFP OV6^+^ cells of two ESCC cell lines. Data are shown as the mean ± SD, *P < 0.05, **P < 0.01. **f** Tumour spheroid formation assay indicated that LV-PRMT1 OV6^+^ cells were able to generate an increased number and size of primary and secondary spheroids compared with the LV-GFP group in ECA109 and TE1 cell lines (scale bar = 100 μm). Data are shown as the mean ± SD *P < 0.05, **P < 0.01

Our previous experimental data indicate that OV6^+^ cells may represent a potential TIC population in ESCC^10^. In this study, we found that PRMT1 messenger RNA (mRNA) and protein expression levels were elevated in magnetically sorted OV6^+^ cells from cultured adherent ECA109 and TE1 cells (Fig. 2a). Similarly, increased levels of PRMT1 expression were also detected in the magnetically sorted OV6^+^ cells isolated from ESCC spheroids (Fig. 2b). When these cultured spheroids were seeded back into adherent conditions, the expression of PRMT1 was correspondingly decreased (Fig. 2c). Taken together, these results indicate that the expression level of PRMT1 is elevated in the esophageal TIC subpopulation.

**Fig. 3:**
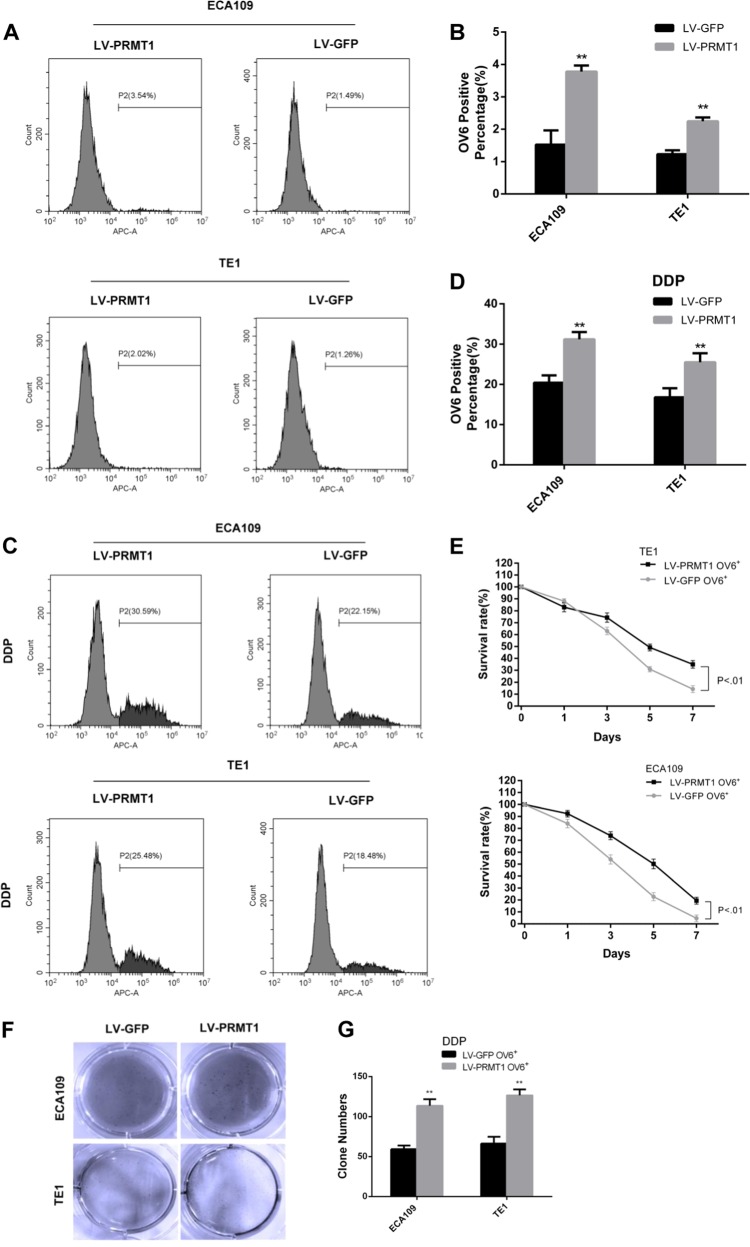
PRMT1 enhances chemoresistance of esophageal TICs. **a**, **b** Flow cytometric analysis showed that the percentage of OV6^+^ cells was significantly increased in LV-PRMT1 cells compared with LV-GFP cells. Data are shown as the mean ± SD, *P < 0.05, **P < 0.01. **c**, **d** Flow cytometric analysis revealed that the standard 4 days of chemotherapy with cis-platinum (2.5 g/ml) augmented the percentage of OV6^+^ subpopulation in ECA109 and TE1 cells. Data are presented as mean ± SD *P < 0.05, **P < 0.01. DDP, cis-platinum. **e** Cyclocystokinin-8 assay (CCK8) indicated that PRMT1 overexpression enhanced the cell viability of ECA109 and TE1 OV6^+^ cells in the presence of cis-platinum (0.6 g/ml). Data are shown as the mean ± SD, *P* < 0.01. **f**, **g** Increased ability of colony formation was also observed in LV-PRMT1 groups compared with LV-GFP groups after treated with cis-platinum (0.6 g/ml) for 14 days. Data are presented as the mean ± SD *P < 0.05, **P < 0.01. All experiments were performed in triplicates

The increased expression of PRMT1 in OV6^+^ cells suggests a crucial role of PRMT1 in esophageal TICs. Therefore, we next tested the role of PRMT1 in the maintenance of stem cell-like properties. We applied lentiviral system contains PRMT1 (LV-PRMT1) to infect ECA109 and TE cells and used lentiviral-luciferase-treated cells (LV-GFP) as a control. The infection and expression efficiency of PRMT1 was verified by immunoblotting (Fig. 2d). Compared with the control group, OV6^+^ cells magnetically sorted from LV-PRMT1 treated ECA109 cells, and TE1 showed an increase in mRNA expression levels of a series of known TICs markers, such as OCT4, CD133, CD44 and so on (Fig. 2e). Consistently, flow cytometric analysis also indicated that over-expression of PRMT1 led to an expansion of the OV6^+^ Eca109 and TE1 cells (Fig. 3a, b).

Esophageal TICs have the ability to form spheroids in vitro, which represents their self-renewal capacity^29^. Assuming that a tumour spheroid formation assay is an important approach for enriching TICs, we examined the impact of PRMT1 on tumour self-renewal ability. As shown in Fig. 2f, significant increases in the number and size of primary and secondary spheroids were detected in OV6^+^ LV-PRMT1 ECA109 and TE1 cells, compared with the OV6^+^ LV-GFP groups. These data suggest that overexpression of PRMT1 enhanced the self-renewal capacity of esophageal TICs.

One of the most important properties of TICs is their ability to resist chemotherapy. To test this hypothesis, flow cytometry was used to compare the percentage of OV6^+^ ESCC cells between the LV-PRMT1 and LV-GFP groups in ECA109 and TE1 cells after 4 days of cis-platinum treatment. As anticipated, cis-platinum enriched more chemo-resistant OV6^+^ ESCC cells in the LV-PRMT1 groups than the LV-GFP groups (Fig. 3c, d). Moreover, overexpression of PRMT1 enhanced the cell viability (Fig. 3e) as well as colony formation capability (Fig. 3f, g) of ECA109 and TE1 OV6^+^ cells in the presence of cis-platinum.

### Overexpression of PRMT1 promotes tumorigenicity of OV6^+^ ECA109 cells in NOD/SCID mice

**Fig. 4:**
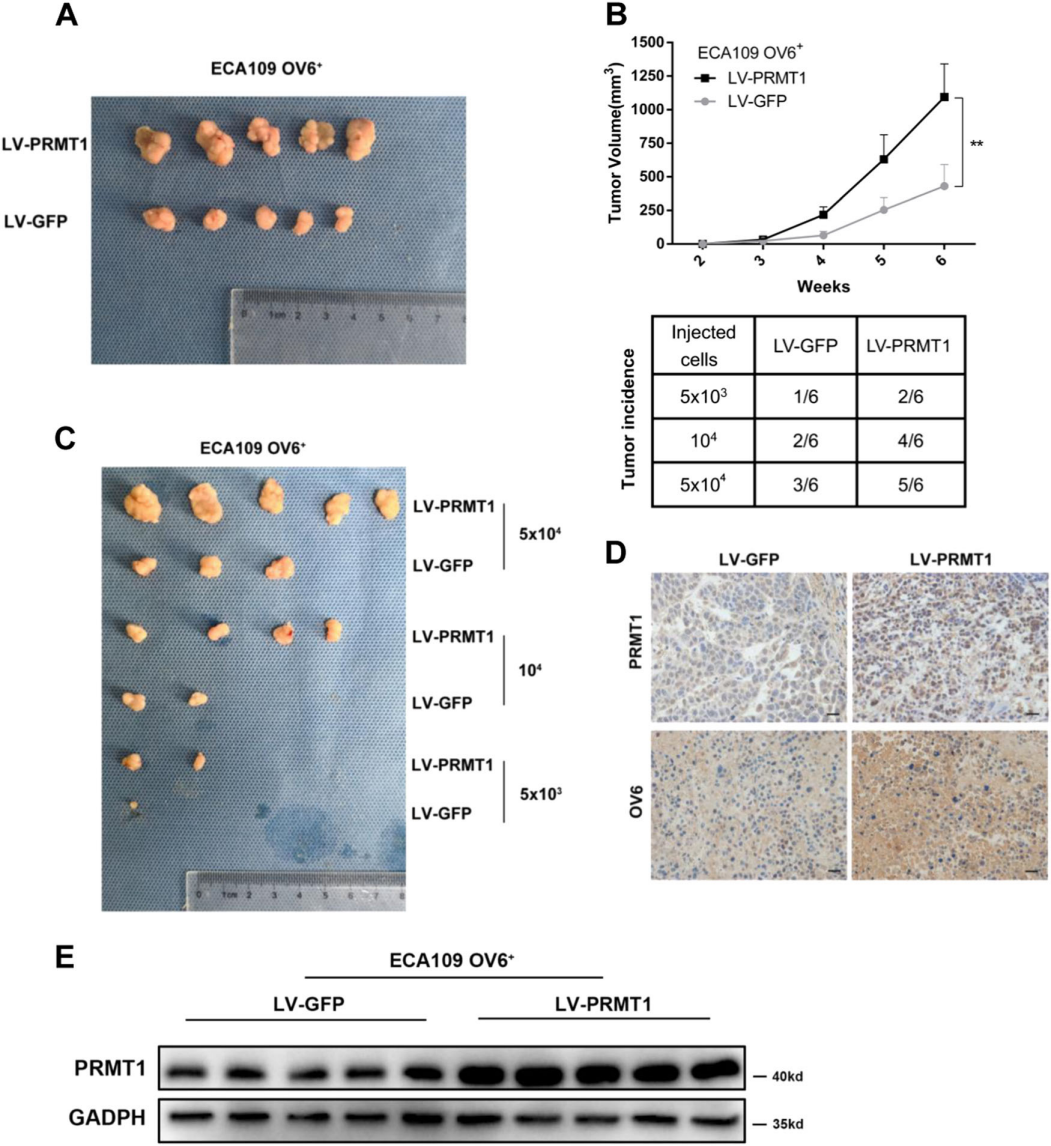
Overexpression of PRMT1 promotes the tumorigenicity of OV6^+^ ESCC cells in vivo. **a**, **b** Representative tumours from mice injected with 5 × 10^4^ LV-PRMT1 or LV-GFP ECA109 OV6^+^ cells after 6 weeks are presented. Tumour volumes from each group were measured weekly. **c**, **b** Tumour incidence in mouse xenografts injected with LV-PRMT1-or LV-GFP-ECA109. NOD/SCID mice were injected with the indicated number of LV-PRMT1 or LV-GFP-ECA109 OV6^+^ cells and tumour incidence in the mouse xenografts were obtained after 28 days. **d** Immunohistochemistry analysis showed the PRMT1 and OV6 expression on mouse subcutaneous tumours inoculated after 6 weeks. Representative image is shown (scare bar = 20 µm). **e** Western blot analysis revealed that the exogenous PRMT1 expression level was elevated in subcutaneous mouse tumours

Our previous data suggested that OV6^+^ ESCC cells, compared with their OV6^−^ counterparts, exhibited an increased capacity to form tumours in vivo^10^. To investigate the impact of PRMT1 on the tumorigenicity of ESCC stem cells, we injected magnetically sorted OV6^+^/PRMT1 cells and OV6^+^/GFP cells into the subcutaneous tissue of NOD/SCID mice. As shown in Fig. 4a, b upper panel, overexpression of PRMT1 significantly enhanced tumour growth (6 weeks). Furthermore, a difference in tumorigenicity was also observed between these two subpopulations (Fig. 4b lower panel, Fig. 4c). Consistently, increased OV6 and PRMT1 expression levels were also observed in the LV-PRMT1 group (Fig. 4d, e). Therefore, these results indicate that PRMT1 expression enhanced the tumorigenicity of OV6^+^ ESCC cells in vivo.

### Knockdown of PRMT1 reduces tumorigenicity of esophageal TICs

**Fig. 5:**
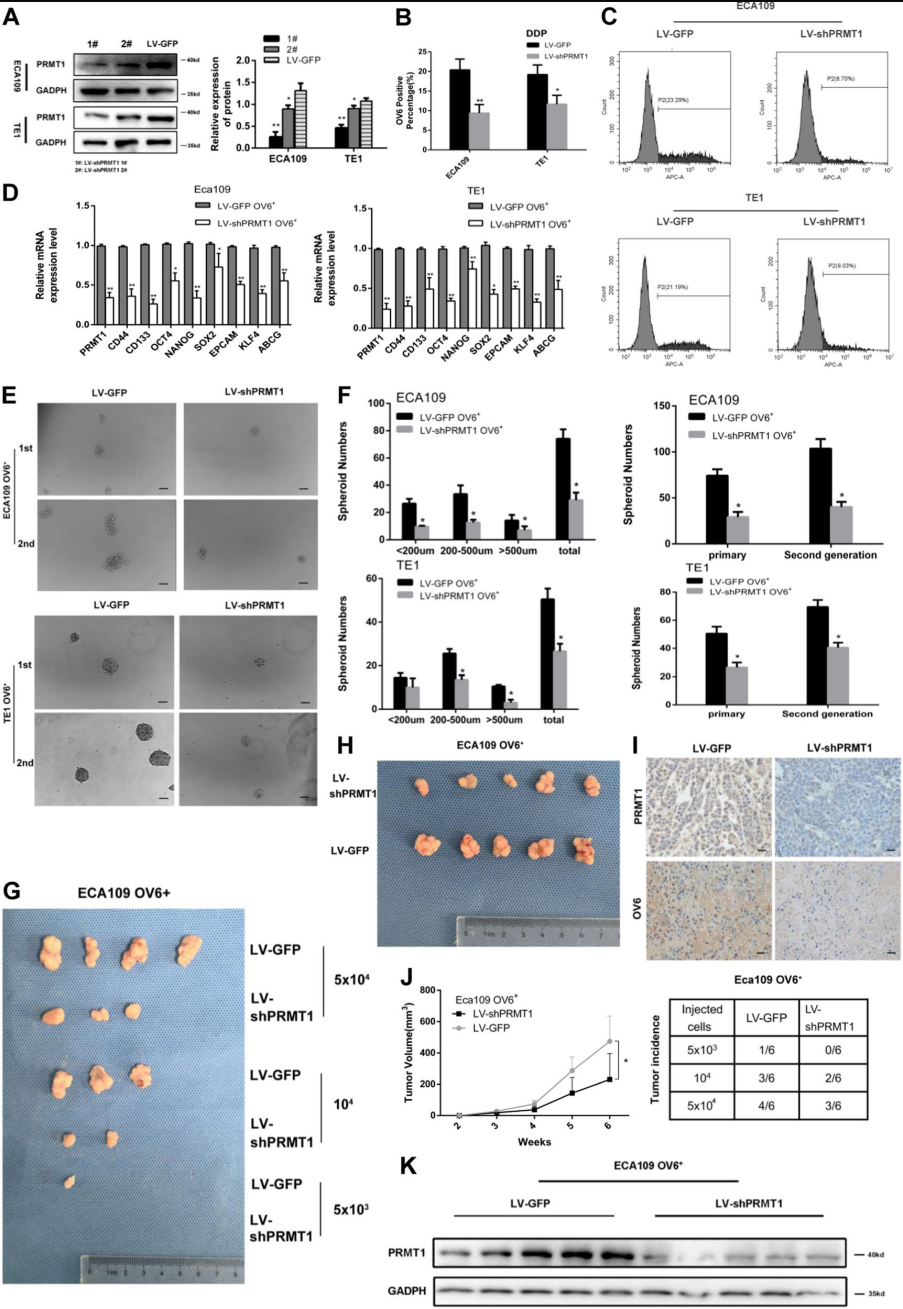
Knockdown of PRMT1 attenuates tumour initiating cell-like properties of TICs. **a** Western blot analysis showed the stable knockdown of PRMT1 by infection with lentivirus LV-shPRMT1 1# and 2# compared with the LV-GFP group. **b**–**f** qRT-PCR, flow cytometric, tumour spheroid formation assay, respectively, showed that knockdown of PRMT1 lead to down-regulation of stem cell-associated genes, the percentage of OV6^+^ subpopulation and capability of spheroid formation. Data are shown as the mean ± SD, *P < 0.05, **P < 0.01. All experiments were performed in triplicates. **g**, **j** NOD/SCID mice were injected with the indicated number of LV-shPRMT1-or LV-GFP-ECA109 OV6^+^ cells and tumour incidence in mouse xenografts was obtained after 28 days. **h**, **j** Representative tumours from mice injected with 5 × 10^4^ LV-shPRMT1 or LV-GFP ECA109 OV6^+^ cells after 6 weeks are presented. Tumour volumes from each group were also measured weekly. **i** Immunohistochemistry analysis showed the PRMT1 and OV6 expression on mouse subcutaneous tumours inoculated after 6 weeks. Representative image is shown (scare bar = 100 µm). **k** Western blot analysis revealed that exogenous PRMT1 expression level was decreased in subcutaneous mouse tumours

To further demonstrate the effect of PRMT1 mediating tumour stem cell-like properties, we utilized a lentiviral-based knockdown approach to silence PRMT1 expression in ECA109 and TE1 cell line. Western blot analysis indicated the successful knockdown of PRMT1 expression in clones 1^#^ and 2^#^, the former of which had a more efficient knockdown effect (Fig. 5a). qRT-PCR analysis indicated that silencing of endogenous PRMT1 in magnetically sorted ECA109 and TE1 OV6^+^ cells lead to significantly decreased expression of stem cell markers (Fig. 5d). Furthermore, chemo-resistance experimental data also displayed that ECA109/TE1-LV-shPRMT1 cells exhibited a reduction in OV6^+^ population after 4 days of *cis*-platinum treatment (Fig. 5b, c). Similarly, spheroids that were decreased in size and primary and secondary number were also found in LV-shPRMT1 OV6^+^ cells (Fig. 5e, f). In addition, PRMT1 downregulation reduced OV6^+^ subset-induced tumorigenicity and tumour growth (Fig. 5g–k). These data show that PRMT1 might play a critical role in the maintenance of esophageal TICs population.

### PRMT1 promotes expansion of TICs by histone H4R3me2a arginine methylation and significantly alters the transcriptome

**Fig. 6:**
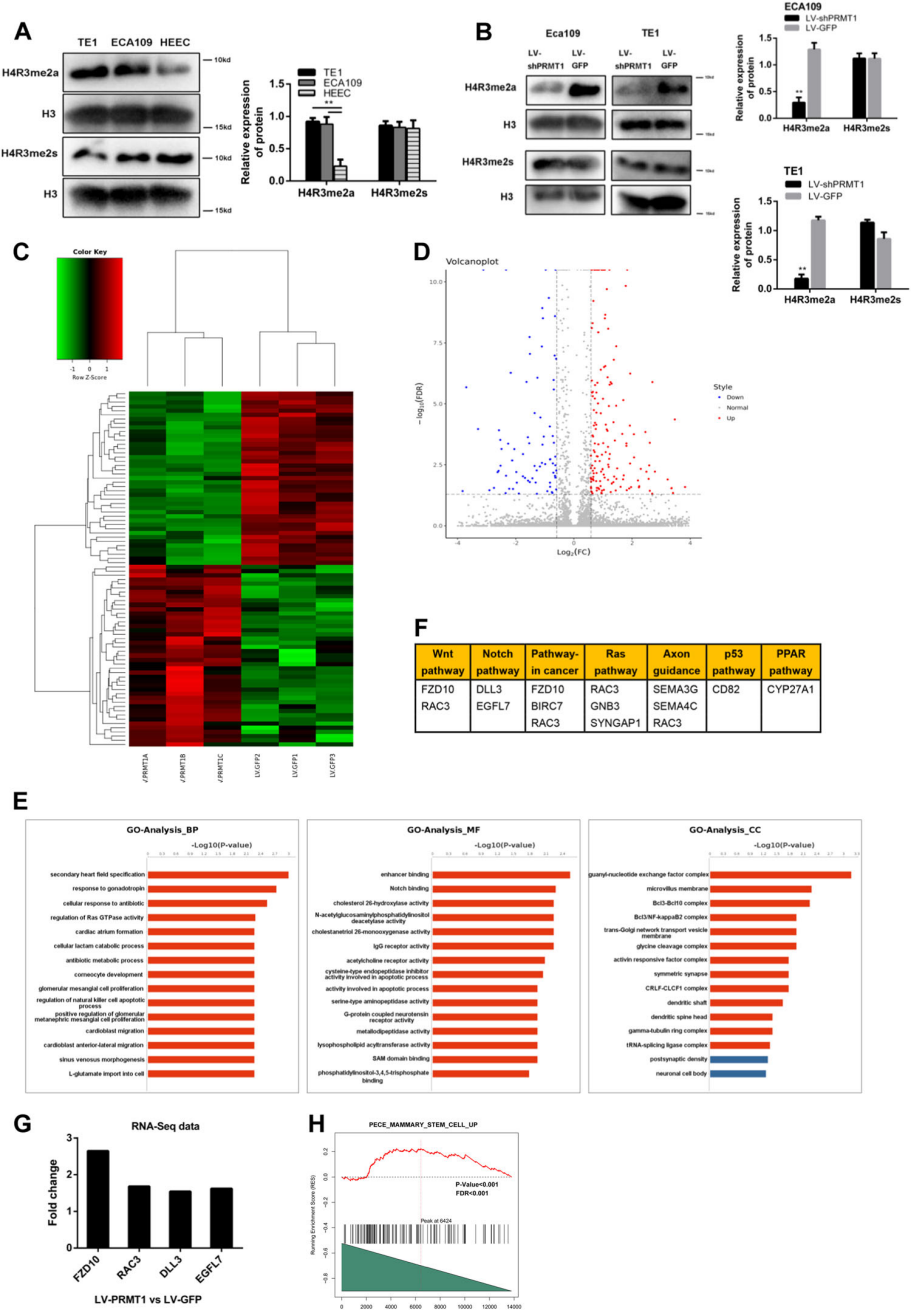
PRMT1 promotes histone H4R3 arginine methylation and alters the transcriptome of ESCC cells. **a** Western blot analysis revealed the histone H4R3 symmetric and asymmetric dimethylation expression levels in HEEC cells compared with ECA109 and TE1 cells. **b** Western blot analysis revealed that histone H4R3 symmetric and asymmetric dimethylation expression levels in the LV-shPRMT1 and LV-GFP groups. **c**, **d** Heatmap and volcano plot of differentially expressed transcripts. **e** Gene ontology (GO) analysis based on all identified transcripts. *P*-value of GO analysis is listed for each category. *P*-value of pathway is colored in red (*P* < 0.05) and blue (0.05 < *P* < 0.1). **f** List of genes upregulated by RNA-Seq analysis in ECA109 cells upon PRMT1 overexpression divided by KEGG pathway. **g** Gene expression fold change of Wnt and Notch pathway from RNA-Seq data. **e** GSEA indicating significant correlations between PRMT1 mRNA expression and stemness-related gene signatures

Previous studies have shown that PRMT1 could catalyse histone H4R3 methylation and promote transcription activation of downstream target genes, in a process that might regulate different cellular functions and phenotypes^30^. In this study, we probed HEEC, ECA109 and TE1 extracts for PRMT1 and the methylation status of their substrates. As shown before, ECA109 and TE1 cells had elevated PRMT1 compared with HEEC cells (Fig. 1c) and had increased H4R3me2a, but not H4R3me2s (Fig. 6a). Next, we compared histone H4 methylation between LV-shPRMT1 and LV-GFP in ECA109 and TE1 cell lines. As shown in Fig. 6b, downregulation of PRMT1 resulted in much lower levels of H4R3me2a histone PTMs, implying that this process might contribute to the maintenance of stem cell-like properties. Since the decreased histone methylarginine levels were observed, we applied RNA-seq to profile the transcriptome in PRMT1 overexpression ECA109 cells versus control ECA109 cells. A total of 249 genes were identified to be differentially expressed and were analyzed to characterize potential biological process, molecular function and pathways (Fig. 6c, d). The GO analysis revealed that Notch binding correlated genes were upregulated by PRMT1 overexpression, including DLL3 and EGFL7 (Fig. 6e, g). Moreover, up-regulated differentially expressed genes related to initiation and progression of cancers were listed in Fig. 6f according to KEGG pathways. Known to regulate self-renewal and stemness properties, the Wnt/β-catenin signal pathway correlated genes were significantly up-regulated in the PRMT1 overexpression cells, including FZD10, RAC3 (Fig. 6f, g). We then conducted gene set enrichment analysis (GSEA) of RNA-Seq data using stemness-related gene signatures and showed that TIC related genes were positively correlated with the PRMT1 overexpression ECA109 cells, indicating that overexpression of PRMT1 upregulated TIC related genes (Fig. 6e).

### PRMT1 directly contributes to the regulation of Wnt/β-catenin and Notch signalling pathway in esophageal TICs

**Fig. 7:**
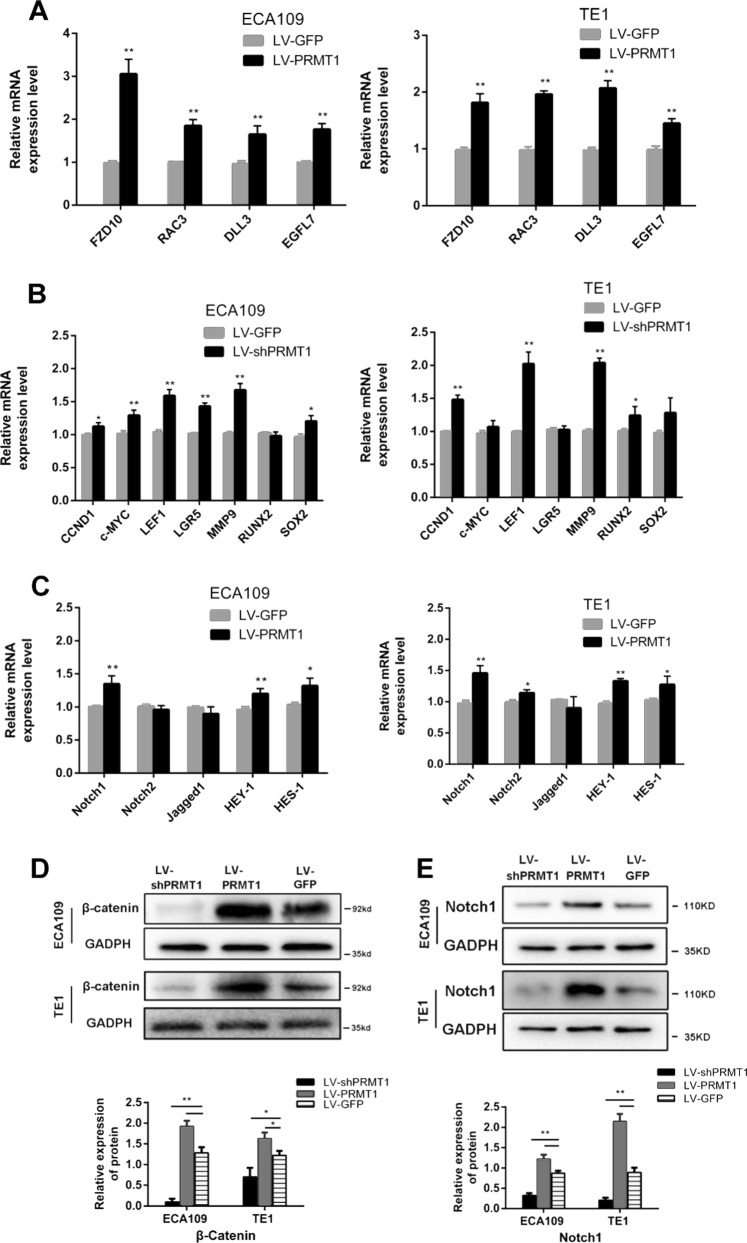
Signaling pathway altered by PRMT1 overexpression in ESCC cells. **a** Four differentially expressed transcripts were validated by qRT-PCR in PRMT1 overexpression ECA109 and TE1 cells. Data is shown as the mean ± SD, *P < 0.05, **P < 0.01. **b**, **c** qRT-PCR analysis for the selected Wnt and Notch target genes in ECA109 and TE1 cells. Data is shown as the mean ± SD, *P < 0.05, **P < 0.01. **d**, **e** Western blot of active (Non-phospho) β-catenin and Notch1 in ECA109 and TE1 with PRMT1 overexpression

To verify the RNA-Seq data of the selected differential expressed genes, qRT-PCR were used to analyse the expression level of DLL3, EGFL7, FZD10 and RAC3 in EAC109 and TE1 cell lines (Fig. 7a). To determine the effect of PRMT1 overexpression on the canonical Wnt/β-catenin signal pathway, we investigated the level of active β-catenin by western blot and found that overexpression of PRMT1 promoted the level of active β-catenin in ECA109 and TE1 cells (Fig. 7d). The qRT-PCR analysis of selected Wnt target genes in the PRMT1 up-regulated ECA109 and TE1 cells further supported the activation of Wnt signaling in these cells (Fig. 7b). We also applied western blot to examine the effects of PRMT1 overexpression on Notch pathway. We found that overexpression of PRMT1 increased the expression of Notch1 and Hes-1, the core molecule involved in Notch pathway, in both ECA109 and TE1 cells (Fig. 7e). The Notch downstream genes were also showed to be up-regulated by qRT-PCR in LV-PRMT1 ECA109 and TE1 cells (Fig. 7c). Taken together, these results indicate that PRMT1 may be responsible for the regulation of Wnt and Notch pathway in esophageal TICs.

## Discussion

Despite advances in therapies in ESCC treatment, the 5 years overall survival rate for ESCC patients still remains poor^31^. Recent studies have reported that TICs played a crucial role in the initiation and progression of many cancers, including ESCC. However, the molecular mechanisms of TICs in mediating tumour invasion and metastasis are not fully understood and needs to be elucidated. In this study, we show for the first time that PRMT1 is elevated in ESCC and plays a crucial role in the promotion of esophageal TICs. We determined that high PRMT1 expression is positively associated with elevated expression of the stem cell marker OV6, and endogenous PRMT1 is also overexpressed in TICs isolated from ESCC cell lines. Moreover, stably PRMT1 expression enhanced stem cell-like properties of ESCC, whereas silenced PRMT1 expression resulted in an opposing effect. More importantly, we also found that PRMT1-catalyzed histone methylation can activate transcription through H4R3 methylation.

PRMT1 was identified as an important oncoprotein that is frequently overexpressed in many tumours, including breast cancer, lung cancer, and colon cancer. In this study, we indicate that PRMT1 is also up-regulated in ESCC and its expression is correlated with aberrant clinicopathological characteristics and prognosis. Previous reports have indicated that PRMT1 promoted the progression of a panel of cancers, such as gastric cancer and breast cancer, by increasing tumorigenicity and invasion potential^32,33^. Our laboratory has also shown that PRMT1 could mediate a series of non-histone proteins methylation and promote epithelial-mesenchymal transition, invasion and metastasis of ESCC (not published). Moreover, It was reported that PRMT1 was found to be the most highly expressed PRMTs in epidermal progenitors and the most downregulated PRMT during differentiation, implying a possible role of PRMT1 in regulating stemness of esophageal squamous cells^34^. Some recent data also suggested that PRMT1 and its binding partner AE9a could promote transcription activation of AE9a-associated genes through enrichment of H4R3 methylation and H3 Lys9/14 acetylation, facilitating the development and self-renewal capacity of leukemia^35^. Additionally, the effects of PRMT1 in maintenance of the stemness of many cells were also observed, including epithelial stem cells, neural stem cells and muscle stem cells^36–38^. However, there are no relevant studies that describe PRMT1’s role in facilitating the development of ESCC through increased self-renewal of TICs.

The process of ESCC development needs to undergo many procedures that begins with a normal squamous epithelium and ultimately to invasive carcinoma. Thus, OV6, the epithelial origin marker, may participate in the progression of ESCC and is used for the isolation of TICs populations. Our previous study has indicated that OV6 could be used as a marker of metastasis ability and self- renewal properties of ESCC^10^. Here, a total of 95 human ESCC samples and different ESCC cell lines were used to evaluate the association of PRMT1 with esophageal TICs. The strong association of high PRMT1 expression in human ESCC with enlargement of OV6 cells, and the increased PRMT1 expressed in magnetically isolated esophageal TICs and spheroid formation supporting our assumption that PRMT1 promotes ESCC initiation and progression via the regulation of tumour initiating cells. Furthermore, stable expression of PRMT1 in isolated OV6^+^ ESCC cells promoted stem cell-like properties, tumour development and chemoresistance. On the contrary, downregulation of PRMT1 exhibited the opposite effects.

As a member of the protein arginine methyltransferase family, PRMT1 is responsible for arginine mono- or asymmetric di-methylation of substrates, which are involved in many cellular processes. For example, the post-translational modification of histones is known to occur on the tail region and contributes to the histone code. PRMT1 is known to methylate histone H4R3 and might contribute to transcriptional activation^39^. However, limited studies focus on the role of PRMT1 in mediating histone methylation throughout tumour progression in patients with ESCC. In the present study, we showed that histone H4R3me2a was elevated in ESCC cells compared with normal esophageal epithelial cells. Moreover, forced PRMT1 expression resulted in a significant increase in H4R3me2a.

The Wnt/β-catenin signaling pathway plays an important role in many biological processes, including stem cell maintenance and cell proliferation^40^. Moreover, the Wnt signaling pathway has been reported to regulate the self-renewal features of TICs in many cancers, including gastric cancer, intestinal cancer^41,42^. Our previous study also showed that the stem-like features of ESCC are maintained by ATG-7 dependent β-catenin regulation. It has also been reported that Notch signaling pathway modulates tumour initiating cell phenotype in breast cancer^43,44^. Here, we applied RNA-seq transcriptome analysis to probe the differentially expressed genes that were altered by enforced PRMT1 overexpression, and some may responsible for the regulation and maintenance stemness properties. In the pathway analysis, we found that overexpression of PRMT1 significantly activated Wnt and Notch signaling pathway. Western blot and qRT-PCR analysis validated that the expression of Wnt and Notch relevant genes increased significantly in LV-PRMT1 ECA109 and TE1 cells. These data exhibit that PRMT1 could improve the tumour-initiating properties of OV6^+^ ESCC cells by promoting Wnt and Notch pathway.

In conclusion, our study has shown an important role of PRMT1 in maintaining stem-like properties of ESCC. The expression of PRMT1 is highly expressed in ESCC and associated with aberrant clinicopathological characteristics. Furthermore, we have identified that PRMT1 is essential for the regulation of esophageal TICs and initiation and progression of ESCC. Therefore, PRMT1 could be responsible for ESCC progression, at least in part, through its regulation of TICs. PRMT1, in combination with OV6 or other TIC markers, provided a novel prognostic marker of HCC. Silencing of PRMT1 in ESCC can significantly suppress ESCC stemness and decreased the self-renewal, tumorigenicity and chemoresistance of OV6^+^ cells via histone H4R3me2a-mediated transcriptome activation. Thus, these results suggest PRMT1 may serve as a therapeutic target to inhibit TICs in ESCC.
